# Transcriptome Analysis Revealed the Paternal Importance to Vegetative Growth Heterosis in *Populus*

**DOI:** 10.3390/plants13162278

**Published:** 2024-08-15

**Authors:** Yuxin Ren, Lixia Wu, Yuhang Zhong, Xinwen Zhao, Meng Xu, Jun Wang

**Affiliations:** 1 State Key Laboratory of Tree Genetics and Breeding, Beijing Forestry University, Beijing 100083, China; ryx18236330386yyy@163.com (Y.R.);; 2 National Engineering Research Center of Tree Breeding and Ecological Restoration, Beijing Forestry University, Beijing 100083, China; 3 Key Laboratory of Genetics and Breeding in Forest Trees and Ornamental Plants, Ministry of Education, Beijing Forestry University, Beijing 100083, China; 4 College of Biological Sciences and Biotechnology, Beijing Forestry University, Beijing 100083, China; 5 Liaoning Provincial Institute of Poplar, Gaizhou 115213, China; 6 College of Forestry, Nanjing Forestry University, Nanjing 210037, China

**Keywords:** heterosis, paternal importance, *Populus*, RNA-Seq, non-additively expressed gene, WGCNA, transcription factor

## Abstract

Parental selection is important for heterosis formation during crossbreeding of *Populus*. However, in poplar hybrids, the effect of parents on vegetative growth heterosis is not well understood. In this study, one female parent (*P*. *simonii* XY4) and two male parents (*P. nigra* OH and *P. deltoides* × *P. nigra* BJLY3#) were used to produce two progenies (Hyb1 and Hyb2). Vegetative growth investigation showed that both Hyb1 and Hyb2 performed heterosis in plant growth and ground diameter. The vegetative growth of hybrids was strongly correlated with the male parents but not with the female parents. The gene expression levels in the hybrids were more biased toward the male parents. In Hyb1 and Hyb2, 51.93% and 45.03% of the expressed genes showed the non-additive effect, respectively, and over 65% of the non-additively expressed genes showed the dominant effect. It is noteworthy that genes of paternal expression dominant effect (ELD_♂) account for the majority of dominantly expressed genes, suggesting the paternal contribution to heterosis. KEGG enrichment analysis indicated that a large number of non-additively expressed genes were enriched in the plant hormone signal transduction pathway. WGCNA analysis showed that MEcyan was significantly correlated with the traits of hybrids, and 12 plant hormone signal transduction pathway genes were enriched in this module. Transcription factors (TFs) MYB88, LHY, and TCP4 may be involved in the regulation of these pathway genes. This finding supported that the male parents play an important role in the formation of vegetative growth heterosis of *Populus*. In addition, the non-additively expressed genes of the signal transduction pathway and the regulation of TFs related to these pathway genes may be one of the reasons for the generation of heterosis.

## 1. Introduction

Heterosis is a common biological phenomenon in nature, which shows that hybrids are superior to the parents in growth, fertility, biomass, stress resistance, and quality [1,2]. Crossbreeding aiming at the production of heterosis has attracted more attention in plant breeding for more than one century. In agriculture, many traits of different crops have been improved by exploiting heterosis [3]. The yield of hybrid rice is 10–20% higher than that of inbred parental varieties [4]. In soybean, two F_1_ hybrids demonstrated significant mid-parent heterosis for node number per stem, pod number per stem, seed number per plant, and the 100-seed weight [5]. In forestry, the utilization of hybrids has brought significant economic benefits to industrial development [6]. In *Eucalyptus camaldulensis* × *E*. *tereticornis*, the content of total lignin and cellulose showed 7.13% and 7.53% advancement compared to parents [7].

Selecting the right parents is one of the key factors in generating favorable progenies with desired traits [8,9,10]. In agriculture, reasonably selected superior parents can enhance some traits in hybrids. In *Brassica napus* L., Xing et al. [11] found that the thousand seed weight of hybrids and parents was mainly controlled by the maternal genotype, and the maternal effect was 58.22% higher than the paternal effect, while the seed yield per plant of hybrids and parents was mainly controlled by the paternal genotype, and the paternal effect was 3.38% higher than maternal effect. In forestry, parental selection is equally important for crossbreeding. In *Hevea brasiliensis*, the average rubber yield of the hybrids with RRII105 as the female parent was higher than the hybrids with RRII105 as the male parent, with 5.7 kg tree^−1^year^−1^ and 4.7 kg tree^−1^year^−1^, respectively, which showed that a female parent with high yield potential is required for the rubber yield improvement in hybrids [12].

At the transcriptional level, changes in gene expression lead to alterations in biological regulatory networks, which can affect heterosis [13]. According to the differences in gene expression between parents and hybrids, gene expression patterns can be divided into additive expression and non-additive expression patterns [14]. Many studies have shown that heterosis in most plant hybrids is due to the non-additive pattern of genes. For instance, Zheng et al. [15] concluded that non-additive action plays a major role in tea volatile heterosis. The same case was observed in *Arabidopsis* hybrids [16] and soybean hybrids [17]. However, in a few cases, this phenomenon was also reported to arise from additive gene expression levels [18].

*Populus* is the most widely distributed and adaptable tree species in the world [19,20,21]. Poplar interspecific and intersectional hybrids showed stronger growth vigor, greater adaptability, fertility, resistance, and other traits as compared to parents [22]. Pearce et al. [23] obtained F_1_ hybrids by using *P*. *deltoides* × *P*. *trichocarpa* and found that F_1_ displayed heterosis for primary growth. In addition, Ren et al. [22] found that the distributions of leaf size traits were biased toward the male parent in *P. simonii* × *P. nigra*, and the heritability estimates of male parents were greater than female parents, which was above 0.56 and 0.17, respectively. Therefore, the questions about how parents are selected during crossbreeding of poplar and how parental selection affects the growth performance in hybrids have been a major concern of the breeders.

In this study, the plant height and ground diameter of two hybrids (Hyb1 and Hyb2) that shared the same female parent (*P. simonii* XY4) and two different male parents (*P. nigra* OH and *P. deltoides* × *P. nigra* BJLY3#) were compared to demonstrate the different heterosis performances. Comparative transcriptome analysis of young leaves of the hybrids and their parents was further performed to reveal the gene expression patterns. The purpose of this study was to explore the importance and contribution of male parents to the formation of heterosis in poplar. The basis of gene expression for heterosis formation derived from male parents was explained, and a theoretical basis for parental selection during crossbreeding was provided.

## 2. Results

### 2.1. Heterosis in Plant Height and Ground Diameter

**Figure 1:**
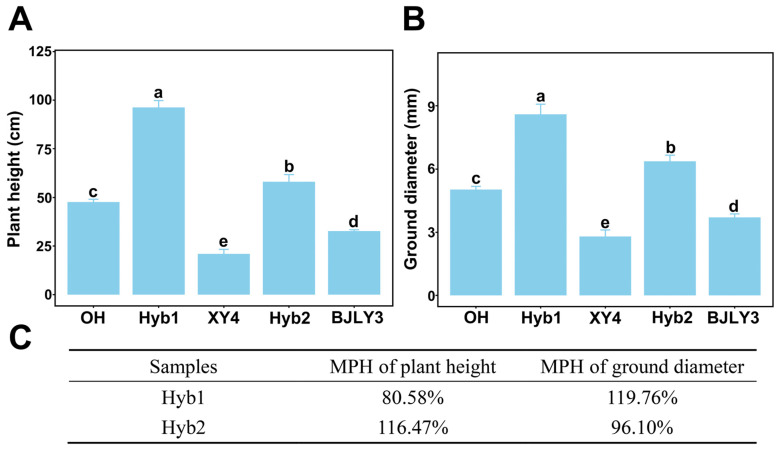
Comparison of the hybrids and their parents on plant height (**A**) and ground diameter (**B**). Hyb1: hybrid1; Hyb2: hybrid2; OH: *P*. *nigra* OH; XY4: *P*. *simonii* XY4; BJLY3: *P*. *deltoides* × *P*. *nigra* BJLY3#. Differences are analyzed by using multiple comparisons. Different lowercase letters represent significant differences between samples (*p* < 0.05). (**C**) The mid-parent heterosis (MPH) of plant height and ground diameter in hybrids.

We compared growth characteristics (plant height and ground diameter) of cutting seedlings of selected hybrids (Hyb1 and Hyb2), female parent (*P. simonii* XY4) and male parents (*P. nigra* OH and *P. deltoides* × *P. nigra* BJLY3#). The selected hybrids in all combinations showed significantly enhanced growth in plant height and ground diameter compared to their corresponding parents (Figure 1A,B). The Hyb1 was the tallest (96.22 ± 3.55 cm) and the thickest (8.60 ± 0.47 mm) among the samples. The OH was the parent with the best growth (47.65 ± 1.41 cm in plant height and 5.03 ± 0.14 mm in ground diameter). By calculating the mid-parent heterosis (MPH) of the hybrids, we found that the MPH of plant height and ground diameter was 80.58% in Hyb1, and the MPH of plant height and ground diameter was 116.47% and 96.10% in Hyb2, respectively (Figure 1C). The results showed that heterosis on plant height and ground diameter growth was produced by hybridization in all combinations. Pearson’s correlation analysis showed that both plant height and ground diameter of hybrids were significantly correlated with that of the male parents (*r* = 0.75, *p* = 3.531 × 10^−4^ for plant height and *r* = 0.61, *p* = 7.681 × 10^−3^ for ground diameter), but not related with that of the female parent (*r* = 0.10, *p* = 0.68 for plant height and *r* = 0.07, *p* = 0.79 for ground diameter), suggesting that male parents are important for seedling vegetative growth heterosis in these combinations.

### 2.2. RNA Sequencing Data Analysis and qPCR Validation

For each cDNA library, more than 37 million row reads were obtained by the Ion Proton sequencing. After removing adapters and low-quality reads, 34.6–45.8 million clean reads and 50.7–63.4 million total base for different libraries were generated, the base filter is over 93%, and the GC content is around 46% (Table S1). An average of 88.4% of the clean reads was mapped to the reference *P*. *trichocarpa* genome (Table S2).

qPCR was conducted to verify the expression levels of eight genes with high and significantly different expressions in different samples. We found that the expression of these eight genes was consistent with the expression trend in transcriptome results (Figure S1). The qPCR results validated the accuracy and reliability of the obtained transcriptome data.

### 2.3. Identification of DEGs between Hybrids and Parents 

**Figure 2:**
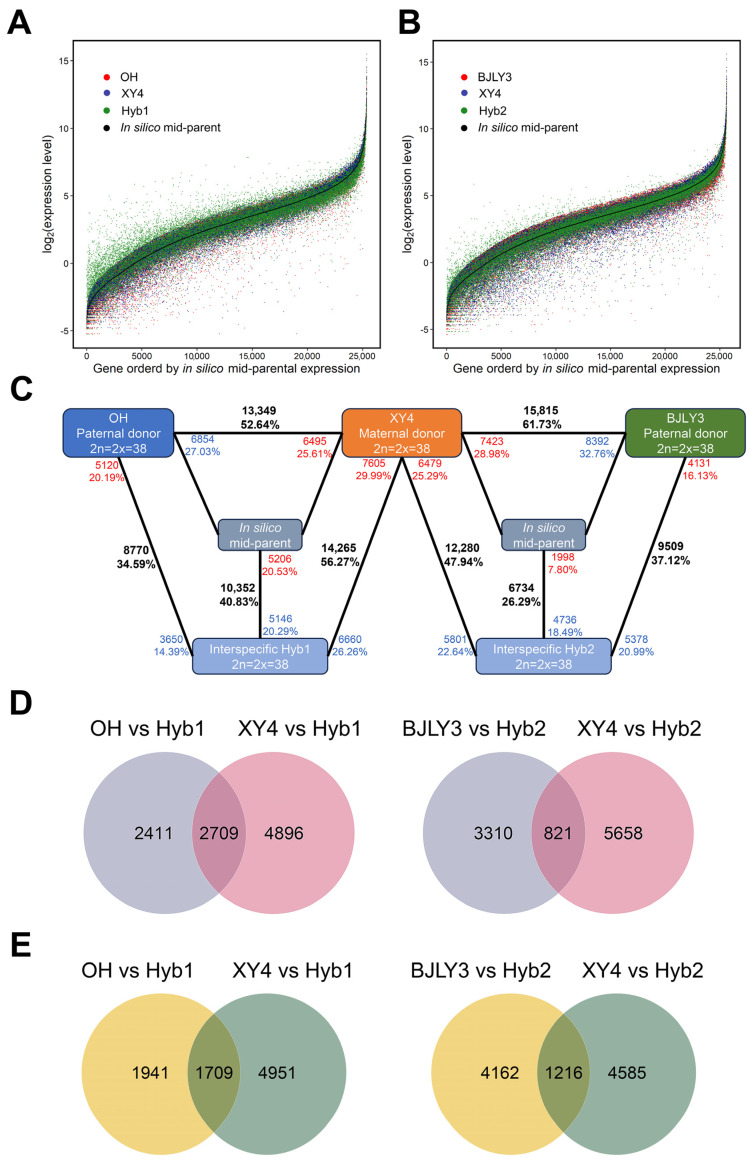
Gene expression levels between parents and hybrids. (**A**) Gene expression levels between the Hyb1 and their parents. Each dot represents a gene, and genes from different individuals are distinguished by colors. (**B**) Gene expression levels between the Hyb2 and their parents. Each dot represents a gene, and genes from different individuals are distinguished by colors. (**C**) Analysis of DEGs in the hybrids and their parents. Black numbers represent all DEGs in the comparative combinations. Red and blue numbers represent up- and down-regulated DEGs in the comparative combinations, respectively. (**D**) Venn diagram of the up-regulated DEGs between the hybrids and their parents. (**E**) Venn diagram of the down-regulated DEGs between the hybrids and their parents.

Among the predicted genes in the *P. trichocarpa* genome, the number of expressed genes were 25,357 and 25,618 for Hyb1 and Hyb2 combinations, which accounted for 61.35% and 61.98% of the total predicted genes, respectively. We plotted the expression level of all the analyzed genes in each combination and in silico mid-parent (Figure 2A,B). Figure 2A,B present a large number of non-overlapped blue and red dots, indicating a high level of gene expression differences between the parents in each combination. Pairwise comparisons of gene expression levels between the parents were performed to assess pre-existing differential gene expression. 52.94–61.73% of expressed genes were differentially expressed between the parents depending on different combinations.

To elucidate gene expression differences between the hybrids and parents, we screened for DEGs using |log_2_(fold change)| ≥ 0.585. The results showed that 8770, 14,265, 9509, and 12,280 DEGs were identified in the four combinations of OH vs. Hyb1, XY4 vs. Hyb1, BJLY3 vs. Hyb2, and XY4 vs. Hyb2, respectively (Figure 2C). Notably, the number of DEGs between the hybrids and the male parents was less than those between the hybrids and the female parent, indicating that gene expression of hybrids was more biased toward the male parents.

In the comparative combinations of parents and hybrids, genes were considered to be up-regulated if their expression in the hybrids was significantly higher than that in the parents; conversely, genes were down-regulated. In view of this, we further classified differentially expressed genes as up- or down-regulated DEGs, conditional on log_2_(fold change) > 0 or <0. The number of down-regulated DEGs was less than the number of up-regulated DEGs in all combinations except for the BJLY3 vs. Hyb2 combination (Figure 2C). The comparative combinations of parents with Hyb1 and Hyb2 include 2709 and 821 up-regulated DEGs and 1709 and 1216 down-regulated DEGs, respectively (Figure 2D,E). In the comparisons between parents and Hyb1, the number of up-regulated DEGs was higher than the number of down-regulated DEGs, whereas the number of up-regulated DEGs was lower than the number of down-regulated DEGs in the comparisons between parents and Hyb2.

### 2.4. Genetic Pattern Analysis of Gene Expression

Genetic differences in gene expression often have an impact on offspring phenotypic variation. It is important to analyze genetic patterns of gene expression to explore the vegetative growth heterosis in the hybrids. In Figure 2A,B, it is clearly shown that some genes in the hybrids (green dots) expressed higher than that in the in silico mid-parents (consecutive black dots) in all combinations, suggesting non-additive expression of these genes in the hybrids.

**Figure 3:**
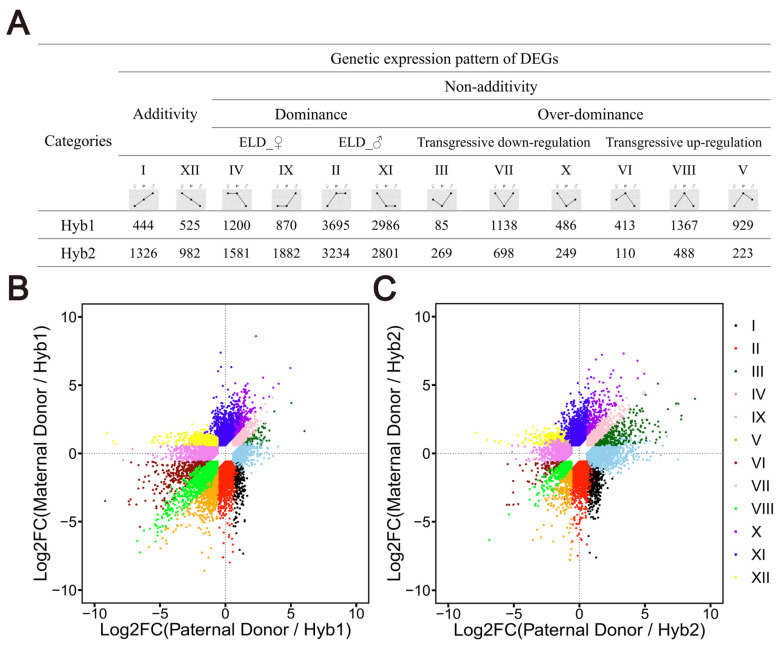
Genetic pattern analysis of gene expression. (**A**) Genetic expression pattern of Hyb1and Hyb2. (**B**) The gene expression groups in Hyb1. (**C**) The gene expression groups in Hyb2. Each point represents a single gene, and different colors represent different groups.

To explore and categorize the gene expression alterations, we classify the genes of hybrids into 12 possible expression patterns (I–XII). In XII and I, the gene expression of the hybrids was intermediate between the parents and had the additive effect; in IV and IX, the gene expression of the hybrids was consistent with the female parent and was maternally biased; in II and XI, the gene expression of the hybrids are consistent with the male parent, and was paternally biased; in III, VII, and X, the gene expression of the hybrids is lower than that of the parents, and was transgressive down-regulation; in VI, VIII, and V, the gene expression of the hybrids are higher than that of the parents, and was transgressive up-regulation. The additively expressed genes in Hyb1 and Hyb2 were 969 (3.82% of the total genes) and 2308 (9.01% of the total genes), respectively, and the non-additively expressed genes were 13,169 (51.93% of the total expressed genes) and 11,535 (45.03% of the total expressed genes), respectively (Figure 3). The non-additive effect can be divided into the dominant and over-dominant effects. The number of dominantly expressed genes in Hyb1 and Hyb2 were 8751 and 9498, accounting for 66.45% and 82.34% of the non-additively expressed genes, respectively. The number of over-dominantly expressed genes were 4418 and 2037, accounting for 33.55% and 17.66% of the non-additively expressed genes, respectively. The dominant effect could be further subdivided into maternal expression dominant effect (ELD_♀) (2070 genes in Hyb1; 3463 genes in Hyb2) and paternal expression dominant effect (ELD_♂) (6681 genes in Hyb1; 6035 genes in Hyb2); the over-dominant effect could be further subdivided into transgressive up-regulation (2709 genes in Hyb1; 821 genes in Hyb2) and transgressive down-regulation (1709 genes in Hyb1; 1216 genes in Hyb2). The results showed that the genetic pattern of gene expression of the hybrids belonged more to II and XI, indicating that the hybrids were biased toward the male parents, which was consistent with the results of phenotypic and DEG analysis.

### 2.5. GO and KEGG Enrichment Analysis of Non-Additively Expressed Genes in Hybrids

**Figure 4:**
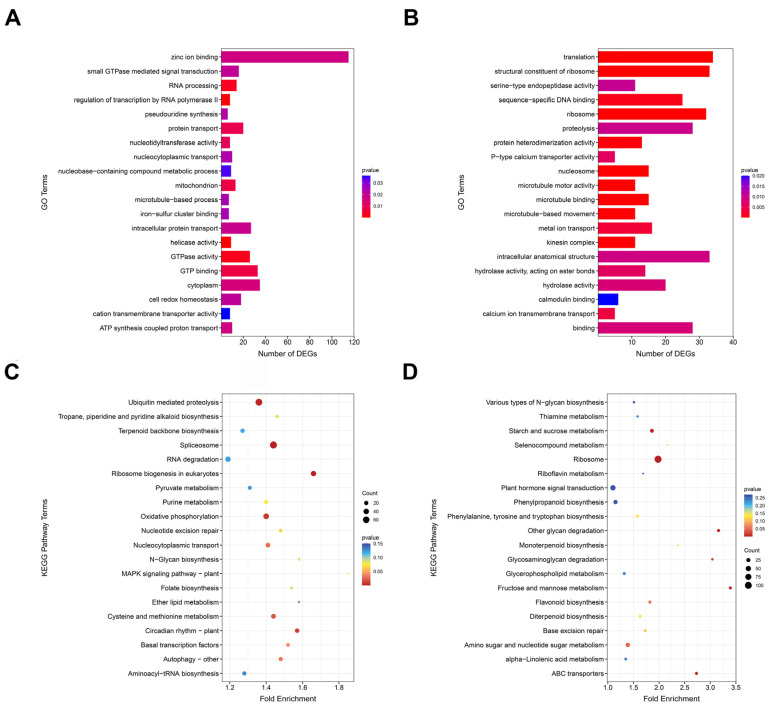
The plots of GO and KEGG enrichment analysis Hyb1. (**A**) The top 20 GO terms of dominantly expressed genes. (**B**) The top 20 GO terms of over-dominantly expressed genes. (**C**) The top 20 KEGG pathways of dominantly expressed genes. (**D**) The top 20 KEGG pathways of over-dominantly expressed genes.

GO and KEGG enrichment analysis was performed for the non-additively expressed genes in the hybrids, and the top 20 GO Terms and KEGG pathways were used for mapping. In Hyb1, we performed the GO enrichment analysis by using dominantly expressed genes and found that some genes were mainly significantly enriched in helicase activity, GTPase activity, and GTP binding, and others were enriched in photosynthesis, signal transduction and photosystem II (Figure 4). Over-dominantly expressed genes were significantly enriched in translation and FMN binding; others were also enriched in signal transduction, photosystem II, and metabolic processes. KEGG enrichment analysis showed that dominantly expressed genes were significantly enriched in circadian rhythm-plant and oxidative phosphorylation, and over-dominantly expressed genes were significantly enriched in ABC transporters and starch and sucrose metabolism.

In Hyb2, GO functional annotation results showed that dominantly expressed genes were significantly enriched to the GO Terms of binding, FMN binding, and membrane, and over-dominantly expressed genes were significantly enriched in the metabolic process, lignin catabolic process. KEGG enrichment analysis showed that dominantly expressed genes were significantly enriched in starch and sucrose metabolism. Over-dominantly expressed genes were enriched in flavonoid biosynthesis (Figure S2).

Notably, the non-additively expressed genes in all two hybrids were enriched in plant hormone signal transduction, photosynthesis, plant-pathogen interaction, and a higher number of dominantly expressed genes in the two hybrids were enriched in plant hormone signal transduction, circadian rhythm-plant, photosynthesis-antenna proteins, photosynthesis, starch and sucrose metabolism, and plant-pathogen interaction. Phytohormones and photosynthesis have important effects on plant growth; therefore, these pathway genes may be important factors that influence poplar vegetative growth heterosis.

### 2.6. Weighted Co-Expression Network Analysis

**Figure 5:**
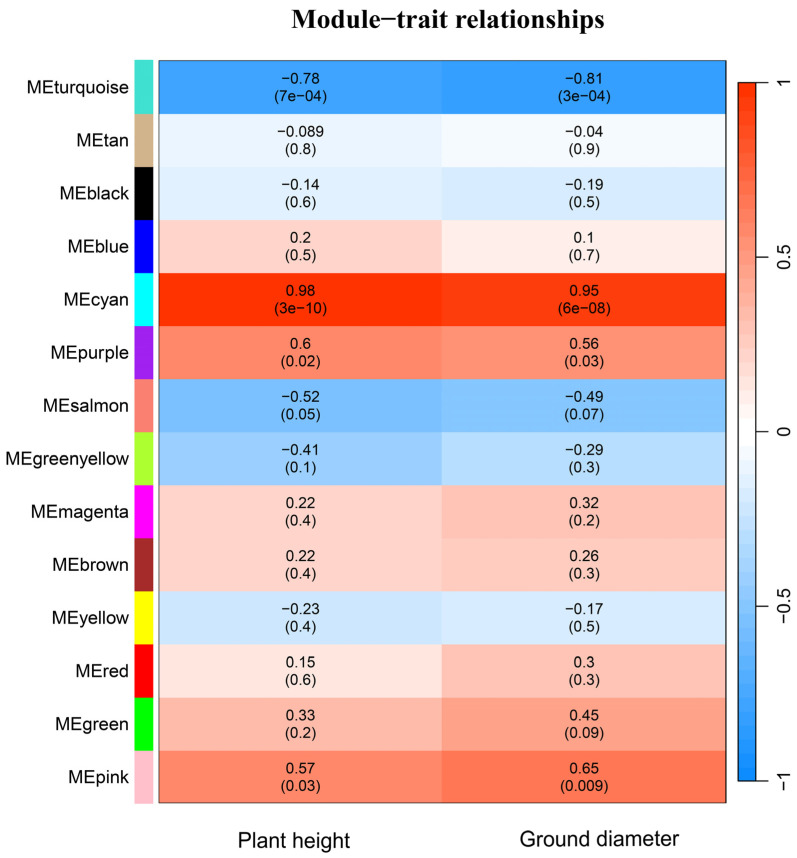
Correlation heat map between phenotype and modules by WGCNA. The horizontal coordinate in the figure represents the traits, and the vertical coordinate represents co-expression modules, which are distinguished by different colours. The numbers in each cell are Pearson correlation coefficients and significance coefficients (the first row of numbers are Pearson correlation coefficients, and the second row of numbers in parentheses are significance coefficients). The colour of the cells is determined by the Pearson correlation coefficient, with red representing a positive correlation and blue representing a negative correlation.

The transcriptome sequence data of two hybrids and three parents, in combination with their phenotypic data, were used for Weighted correlation network analysis (WGCNA) to identify co-expression modules related to poplar vegetative growth heterosis. After clustering, genes with similar expression patterns were clustered into the same module. A total of 14 modules were identified, including MEturquoise, MEtan, MEblack, MEblue, MEcyan, MEpurple, MEsalmon, MEgreenyellow, MEmagenta, MEbrown, MEyellow, MEred, MEgreen, MEpink (Figure 5). Among these modules, MEsalmon had the largest number of module genes, with 8245 genes, while MEred had the smallest number of module genes, with two genes. Two modules, MEcyan (plant height, *r* = 0.98, *p* < 0.001; ground diameter, *r* = 0.95, *p* < 0.001) and MEturquoise (plant height, *r* = −0.78, *p* < 0.001; ground diameter, *r* = −0.81, *p* < 0.001) was highly correlated with plant height and ground diameter.

A KEGG enrichment analysis of genes in the MEcyan module showed that these genes were mainly involved in plant hormone signal transduction, starch and sucrose metabolism, plant-pathogen interaction, and circadian rhythm-plant. KEGG enrichment analysis of genes in the MEturquoise module found that these genes are mainly involved in brassinosteroid biosynthesis, plant hormone signal transduction, plant-pathogen interaction, and circadian rhythm-plant. It is speculated that the genes in these modules may be related to poplar growth heterosis.

### 2.7. Identification of Regulators of Plant Hormone Signal Transduction Pathway

**Figure 6:**
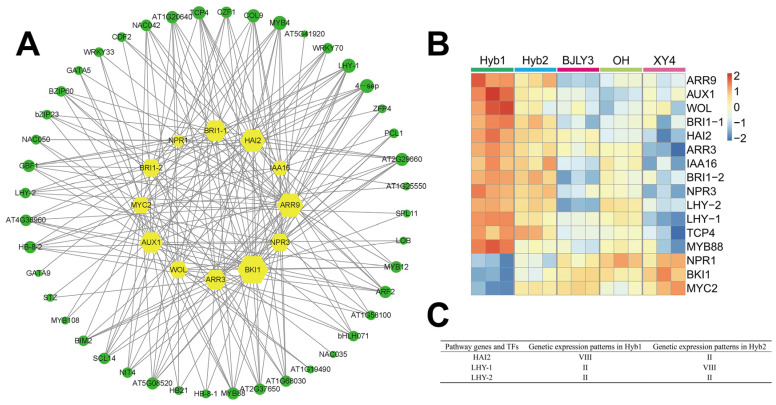
(**A**) Co-expression network between pathway genes (plant hormone signal transduction) and TFs. The green circular nodes represent TFs. The yellow hexagon nodes represent the pathway genes of the plant hormone. The node size is positively correlated with the gene degree value. (**B**) The expression heat map of plant hormone signal transduction pathway genes of MEcyan module and some TFs. (**C**) Genetic expression patterns of some plant hormone signal transduction pathway genes and TFs in hybrids. II, high-paternal expression dominance; VIII, transgressive up-regulation.

In the present study, 63 TFs, representing 26 TF families, were identified in the MEcyan module. Most of the TFs belong to the MYB, NAC, WRKY, and other families. To identify TFs that were significantly associated with the pathway of plant hormone signal transduction, a co-expression network of 12 pathway genes was constructed. The screening threshold for high correlation was set as the absolute value of *r* > 0.8. A total of 216 pairs of corresponding relationships between 12 genes and 44 TFs were identified (Figure 6A). TFs associated with the genes in the network were further analyzed, and it was found that TFs such as MYB88, LHY, and TCP4 had high connectivity with the pathway genes. Therefore, we compared the expression of 12 pathway genes and 4 TFs between hybrids and parents. We found that among the pathway genes, *brassinosteroid insensitive 1* (*BRI1*) and *BRI1 kinase inhibitor 1* (*BKI1*) were highly correlated with TFs, in which the expression of *BKI1* was down-regulated expression in the hybrids, whereas the two *BRI1* genes were up-regulated expression in the hybrids (Figure 6B). *Highly ABA-induced PP2C gene 2* (*HAI2*) was up-regulated expression in the hybrids (Figure 6B,C). In addition, the expression of TFs in the hybrids differed from that of the parents.

## 3. Discussion

The utilization of heterosis has always been the focus of poplar breeding. In the study, two hybrids with the same female parent had significantly better growth traits than their parents, including plant height and ground diameter. These results are consistent with previous findings in other species. For example, Azad et al. [24] observed that 17 rice hybrids demonstrated significantly positive heterosis in plant height, and the greatest mid-parent heterosis value was 46.66%. In the soybean hybrid Jilin 38 × Jilin 47, the index of mid-parent heterosis and better-parent heterosis in seedling height were 25.01% and 20.57%, respectively [25]. In addition, the plant height and ground diameter of the two hybrids were significantly correlated with their respective male parents but not with their female parents, indicating that the male parents had an important influence on the vegetative growth of the hybrids in the two crossing combinations. However, in the hybrids of *Miscanthus sinensis* and *M*. *lutarioriparius*, the traits of plant height, stem diameter, tiller number, and stem node number were mainly influenced by the female parents [26]. The differences in parental contributions may be varied depending on the species.

Rational selection of parents is a key factor in obtaining strong hybrids [27,28]. In the rice hybrids, Jing-Liang-You-Hua-Zhan and Long-Liang-You-Hua-Zhan, the expression patterns were more similar to the female parent, as demonstrated by the fact that the number of DEGs between the hybrids and the female parents was less than between the male parent [29]. However, in *H*. *brasiliensis*, the gene expression level of the hybrid progeny RY7-20-59 was more similar to its male parent PR107 [30]. In the hybrid rice Liang-You-Pei-Jiu, the expression profile of panicles highly resembled its male parent, with a correlation coefficient of 0.95 [31]. In *B*. *napus*, Xiong et al. [32] found that the number of DEGs between all hybrids and the male parents was less than that between the hybrids and female parents. In the study, transcriptome analysis revealed that some genes were differentially expressed between the parents and the hybrids, and these DEGs largely contributed to the generation of heterosis in the two hybrids. In addition, the number of DEGs between male parents and hybrids was less than between the female parent and hybrids in all comparative combinations. We found that the two hybrids were biased towards the male parents, both in terms of phenotypes and gene expression levels, which suggests that the male parents have an important influence on the nutritional growth heterosis of hybrids in the two crossing combinations.

With the development of modern technology, a lot of research has been conducted to explain the formation of heterosis at the transcriptional level, and it has been found that non-additively expressed genes are commonly present in hybrids, which include both dominant effect genes and over-dominant effect genes [33,34,35,36]. Some studies have suggested that the over-dominant effect is the key to heterosis. Chen et al. [37] analyzed the transcriptome of the development of young panicles of the rice variety ‘WFYT025’ and found that the over-dominant effect accounted for 63.1%. Therefore, they argued that the over-dominant effect may be the main reason for the heterosis of the grain number. However, most of the genes expressed in Chinese cabbage hybrids are dominant, accounting for 72.83–84.20% of the total genes [38]. In the present study, by analyzing overall gene expression level difference in the hybrids relative to the in silico mid-parent, we found that more than 80% of expressed genes in Hyb1 and Hyb2 showed a non-additive pattern, with the majority of non-additively expressed genes showing a dominant effect, which suggested that the dominant effect genes might influence the vegetative growth of hybrids. Notably, the number of genes in ELD_♂ was overwhelming in all dominantly expressed genes. This result might be the reason why the traits of hybrids were significantly correlated with their male parents.

As important regulators of plant growth, plant hormones play an important role in the process of growth and development in plants [39]. In the present study, we found some non-additive genes in two hybrids enriched in the plant hormone signal transduction pathway. Previous studies have shown that brassinosteroids (BRs) are one class of plant hormones that can produce important effects on plant height [40]. As the binding of BR to *BRI1*, BR signal transduction is activated, whereas in the absence of BR, the negative regulator *BKI1* replaces BR in binding to *BRI1* and inhibits *BRI1* function [41,42]. Secondary growth in plants results in the thickening of the stem, which is also influenced by the regulation of plant hormones [43]. In *Arabidopsis*, Sehr et al. [44] found that jasmonic acid (JA) signaling positively regulated the formation of cambium and identified two cambium regulators, *coronatine insensitive 1* (*COI1*) and *MYC2*, that promoted secondary growth, which are components of the JA signaling pathway [44]. In our study, the expression of *BKI1* in two hybrids was similar to that of the male parents but was up-regulated in the female parent, *BRI1* was up-regulated in the hybrids, and *COI1* was up-regulated in both hybrids as well as the male parents. In addition, genes involved in the regulation of hormones, such as cytokinin, auxin, and abscisic acid, were also differentially expressed in the hybrids and the parents; for example, the PP2C protein is involved in abscisic acid (ABA) signalling. Additionally, functional annotation of paternal expression dominance genes in Hyb1 and Hyb2 showed that 7 and 13 genes were associated with photosynthesis, respectively. It has been reported that the *photosystem II subunit Q-2* (*PsbQ-2*) gene encoded PsbQ proteins and was essential for the stabilization of oxygen-evolving PS II complexes [45], which was up-regulated in hybrids and male parents. These changes in the expression of genes related to plant hormone signal transduction and photosynthesis-related proteins may be one of the reasons for the generation of heterosis in poplar plant height and ground diameter.

The co-expression network was used to explore the regulatory relationship between TFs and pathway genes. In *Arabidopsis*, Xie et al. [46] found that *flp-1 myb88* mutants were deprived of the ability to sense ABA. It has been reported that LHY responds to drought stress by regulating ABA signaling [47]. LHY inhibited ABA biosynthesis by repressing the transcription of 9-cis-epoxycarotenoid di-oxygenase 3 (NCED3) and promoted the expression of ABA-responsive genes [48,49]. *HAI2*, as a negative regulator of ABA signaling, has been demonstrated as a LHY target [49,50]. Challa et al. [51] found that *TCP4* was able to directly activate the expression of *YUCCA5* and regulate downstream regulators of auxin-brassinosteroid signaling to promote cell elongation with the influence of environmental factors. In our study, the co-expression network was used to explore the regulatory relationship between transcription factors and pathway genes. Our results showed that *MYB88*, *LHY*, *TCP4*, and other TFs had high connectivity with pathway genes, and *MYB88* showed transgressive up-regulation (VIII) in Hyb1, *LHY* showed high-paternal expression dominance (II) in two hybrids (Table S4). Moreover, *HAI2*, which was significantly positively correlated with *LHY*, was up-regulated expression in hybrids. Therefore, *LHY* might regulate the expression of *HAI2* in hybrids, thereby affecting the ABA signalling pathways and reducing the inhibitory effect of ABA on plant growth. The change might influence the generation of heterosis in poplar.

In recent years, how to obtain fast-growing poplar trees has been an important breeding objective [52]. Li et al. [53] obtained 117 triploids by high temperature-induced chromosome doubling of female gametes in *Populus*. Huang et al. [54], by comparing diploids and triploids (triploids obtained by colchicine-induced embryo sac chromosome doubling) of poplar trees with the same parents, found that triploids were significantly longer than diploids in fibre length. Similarly, chromosome doubling of male gametes has been reported as an important means for obtaining triploids in poplar trees [55]. According to our results, the influence of male parents in poplar heterosis was preliminarily explored. Subsequently, in poplar breeding, we can consider selecting male parents with excellent traits, and it can be expected to obtain poplar triploid germplasms with more excellent characters by doubling the male gametes and then crossing them with normal female gametes, which combining the ploidy vigour and heterosis.

## 4. Materials and Methods

### 4.1. Plant Materials and Growth Conditions

Two hybrid progenies (Hyb1 and Hyb2) from different interspecific combinations of *Populus* and their parents were used in this investigation. The three parents were *Populus simonii* XY4 (hereafter referred to as XY4), *P. nigra* OH (hereafter referred to as OH), and *P. deltoides* × *P. nigra* BJLY3# (hereafter referred to as BJLY3). The Hyb1 was produced by crossing XY4 (female parent) with OH (male parent); the Hyb2 was produced by crossing XY4 (female parent) with BJLY3# (male parent). The Hyb1 shared the same female parent XY4 with the Hyb2. All plants were grown in the field at the nursery garden of Weixian County, Hebei Province, China. Ten high-growth hybrid genotypes for each combination were selected from the field. All selected genotypes and parents were determined as diploid by flow cytometry. All selected genotypes of the hybrids were grown for one year to obtain annual stem segments. Parents were juvenilized by vegetative propagation to obtain annual stem segments.

Annual stem cuttings of the selected genotypes and parents were planted in peat soil in plastic pots (25 cm × 25 cm in both diameter and depth). One annual stem segment was planted in each pot, with a total of 10 pots per genotype. All plant materials were grown in the greenhouse (28 °C) of Beijing Forestry University under natural light. Five pots of plant material were placed in each row for a total of 10 rows, and each pot was spaced 60 cm. After three months of growth, six pots of uniformly growing plants were selected, and plant height and ground diameter of all plants were measured by using a tape measure and a vernier caliper, respectively. Young leaves from the selected plants were collected and frozen in liquid nitrogen for later use.

### 4.2. Calculation of Mid-Parent Heterosis

Mid-parent dominance (MPH) for plant height and ground diameter was calculated following Zhai et al. [56]. The calculation formula was MPH = (F_1_ − MP)/MP in %, where F_1_ is the mean value of traits in hybrids, and MP is the average performance of traits in two parents.

### 4.3. cDNA Preparation and Ion Porton Sequencing

The six high-growth hybrid genotypes for each combination were divided into three pools consisting of two plants serving as three biological replicates for RNA isolation. Equal amounts of RNA from individuals within each group were pooled to ensure equal representation within each RNA sample. Three plants for each parent and progenies were used as three biological replicates. 

The cDNA libraries for single-end sequencing were prepared using Ion Total RNA-Seq Kit v2.0 (Life Technologies, Carlsbad, CA, USA). Fragment the RNA using RNAse III, then purify and evaluate the fragmented RNA. After hybridization and ligation of the fragmented RNA, reverse transcription was performed to obtain cDNA. The cDNA was purified and amplified, and then the amplified cDNA was also purified and evaluated. The cDNA libraries were performed for sequencing based on commercially available protocols by using the Ion Proton™ system (Life Technologies). Samples were diluted and mixed, and the mixtures were manipulated on the OneTouch 2 instrument (Life Technologies) to prepare and recover the template-positive Ion PI™ Ion Sphere™ Particles (Life Technologies) in accordance with Ion PI™ Template OT2 200 Kit v2.0 (Life Technologies). The mixed template-positive Ion PI™ Ion Sphere™ Particles of samples were enriched by the OneTouch 2 ES station (Life Technologies), then loaded onto the 1 P1v2 Proton Chip (Life Technologies) and sequenced on Proton Sequencers according to Ion PI Sequencing 200 Kit v2.0 (Life Technologies).

### 4.4. Quality Control and Mapping of RNA-Seq

Quality control of transcriptomic sequencing data was accomplished by removing the low-quality reads, such as adaptor sequences, and reads with >5% ambiguous bases (noted as N). Finally, the filtered clean reads were obtained from the raw data for subsequent mapping. The clean reads of each sample were aligned to reference genome *P*. *trichocarpa* v3.0 (https://phytozome.jgi.doe.gov/pz/portal.html, URL (accessed on 18 July 2021)) [57] using the MapSplice program (v2.1.8) [58] under following parameter (-s 22 -p 8 --ins 6 --del 6). Mapping results are presented in Table S2.

### 4.5. Gene Expression Analysis

The uniquely mapped reads for each sample were counted by HtSeq-Counts [59]. The read counts were analyzed with the edgeR package in the R statistical environment [60]. The CPM (counts per Mb) value was used to represent the expression level of each gene. Each combination was analyzed separately. Only genes whose expression could be detected in 9 samples of each combination were left for expression comparisons. Values from the three biological replicates of each parent were averaged for in silico mid-parental expression value.

### 4.6. Identification of Differentially Expressed Genes (DEGs) and Gene Inheritance Patterns

Using |log_2_(fold change)| ≥ 0.585, false discovery rate (FDR) < 0.05 as criteria, we screened for DEGs between parents and hybrids. Based on log_2_(fold change) > 0 or <0, DEGs were further divided into up- or down-regulated genes. According to the method of [61], the genetic patterns of DEGs were divided into three categories: additivity, dominance, and over-dominance, and further subdivided into 12 categories.

### 4.7. Functional Annotation of DEGs

To clarify the related functional terms and pathways of DEGs, the gene ontology (GO) and Kyoto encyclopedia of genes and genomes (KEGG) enrichment analysis of DEGs was implemented using the R package ClusterProfiler (v4.2.1). KEGG is a database resource for understanding high-level functions and utilities of the biological system (http://www.genome.jp/kegg/, URL (accessed on 27 December 2022)). The *p* < 0.05, as a threshold, was considered as significantly enriched.

### 4.8. Gene Co-Expression Network Analysis and Visualization

The R package WGCNA (v4.2.2) is used to build the gene co-expression network [62]. Genes with similar expression patterns were clustered and divided into unified modules. Modules with *p* < 0.05 and |*r*| > 0.7 were designated as highly correlated with the phenotypic traits. The genes within the modules highly correlated with the phenotypic traits were subjected to KEGG enrichment analysis, and the genes enriched to plant hormone signal transduction were extracted for the next step of analysis. Gene co-expression networks were visualized using Cytoscape software (version 3.8.2) [63].

### 4.9. Quantitative Real-Time PCR (qPCR) Analysis

To verify the accuracy of transcriptome data, 8 DEGs with high expression levels (*Potri.002G146300*, *Potri.003G136700*, *Potri.009G052500*, *Potri.010G132000*, *Potri.013G057500*, *Potri.002G180800*, *Potri.011G087200*, *Potri.012G031100*) were validated by quantitative real-time PCR, which using the actin gene (GenBank: EF418792.1) as the internal control. All experiments were performed with the ABI QuantStudio 6 Flex Real-Time PCR system (Applied Biosystems, Foster City, CA, USA). The primers used are shown in Table S3. The 2^−ΔΔCt^ method was used to calculate the relative expression level. SPSS v26.0 was used to analyze the differences in gene expression levels. All processes include three biological repeats.

## 5. Conclusions

In this study, we found that the phenotype and gene expression levels of hybrids were more closely resembling their male parents, and the paternal expression dominant effect was the main expression pattern of the hybrids. Non-additive expression of plant hormone signal transduction pathway genes and the regulation of TF may play an important role in the generation of poplar heterosis. This study explained the importance of male parents to the formation of heterosis at a transcriptional level and provided new insights into the process of parental selection in crossbreeding.

## Data Availability

The datasets generated during the current study are available in the National Genomics Data Center (NGDC) repository under accession numbers CRA015605, CRA015608, CRA015611, CRA015613, and CRA015614.

## Supplementary Materials

The following supporting information can be downloaded at: https://www.mdpi.com/article/10.3390/plants13162278/s1. Figure S1. qPCR validation of the transcriptome data. Figure S2. The plots of GO and KEGG enrichment analysis Hyb2. A–B The top 20 GO terms of dominance and over-dominance, respectively. C–D The top 20 KEGG pathways of dominance and over-dominance, respectively. Table S1. Quality control results of transcriptome sequencing of hybrids and parents in *Populus*. Table S2. Mapping statistics of hybrids and parents in *Populus*. Table S3. Sequences of gene-specific primers for transcriptome data and qPCR detection. Table S4. Genetic expression patterns of plant hormone signal transduction pathway genes and TFs in hybrids.

## Abbreviations

ABA	abscisic acid
*BKI1*	BRI1 kinase inhibitor 1
BR	Brassinosteroid
*BRI1*	Brassinosteroid insensitive 1
*COI1*	Coronatine insensitive 1
DEG	Differentially expressed gene
ELD_♀	Maternal expression dominant effect
ELD_♂	Paternal expression dominant effect
FDR	False discovery rate
GO	Gene ontology
*PsbQ-2*	photosystem II subunit Q-2
*HAI2*	Highly ABA-induced PP2C gene 2
JA	Jasmonic acid
KEGG	Kyoto encyclopedia of genes and genomes
*NCED3*	9-cis-epoxycarotenoid dioxygenase 3
TF	Transcription factor
WGCNA	Weighted gene co-expression network analysis
