# Supplementary material for: Transcriptome Analysis Revealed the Paternal Importance to Vegetative Growth Heterosis in Populus
Source: Plants (Basel). 2024 Aug 15;13(16):2278. doi: 10.3390/plants13162278 (PMC11359908; doi:10.3390/plants13162278)
Supplement: Supplementary file 1 [file plants-13-02278-s001.zip › plants-3043385-supplementary.pdf]

Tables

Table S1. Quality control results of transcriptome sequencing of hybrids and parents in *Populus*

Sample Name	Total Reads_ Before	Total Base_ Before	Total Reads_ After	Total Base_ After	Reads Filter %	Base Filter %	GC%_ Before	GC%_ After
Hyb1_1	45413586	5805033050	42408353	5596075851	0.933825243	0.964004133	46	46
Hyb1_2	42438311	5633692940	39998818	5456878660	0.942516727	0.968614853	46	46
Hyb1_3	45087328	6300196989	42259780	6100582403	0.937287302	0.968316136	46	46
Hyb2_1	43167203	6002702741	40418359	5805865126	0.936321007	0.967208502	46	46
Hyb2_2	42083010	5682213935	39786759	5521414713	0.945435201	0.971701308	46	46
Hyb2_3	39240102	5298356927	36954918	5136910801	0.941764066	0.96952902	46	46
BJLY3_1	42754464	6387701225	40034762	6184802043	0.936387882	0.968235962	46	46
BJLY3_2	43062130	6566629997	39956183	6330758117	0.92787289	0.964080224	46	46
BJLY3_3	49175013	6225239173	45728019	5999428474	0.929903547	0.963726583	46	46
OH_1	45518526	5425811018	40412793	5078798336	0.887831759	0.93604409	47	47
OH_2	45846424	5409897657	40913870	5072370937	0.892411369	0.937609408	46	46
OH_3	46043416	6486232269	42611518	6226608111	0.925463871	0.95997304	46	46
XY4_1	37902277	5410860666	34645083	5170111568	0.914063369	0.955506321	45	46
XY4_2	42091370	6132093842	37713396	5817517387	0.895988798	0.948699993	45	45
XY4_3	40365159	5837955200	36568438	5566690408	0.905940641	0.95353428	46	46

Table S2. Mapping statistics of hybrids and parents in *Populus*

Sample Name	Total Reads_After	Unmapped	Mapped	Mapped Rate	Unique Mapped	Unique Mapped Rate
Hyb1_1	42408353	6058232	36350121	0.857	33562568	0.791
Hyb1_2	39998818	4540627	35458191	0.886	32837469	0.821
Hyb1_3	42259780	5175101	37084679	0.878	34395738	0.814
Hyb2_1	40418359	4039474	36378885	0.9	34454424	0.852
Hyb2_2	39786759	4174661	35612098	0.895	33627875	0.845
Hyb2_3	36954918	3906932	33047986	0.894	31219107	0.845
BJLY3_1	40034762	4930433	35104329	0.877	33231921	0.83
BJLY3_2	39956183	4639410	35316773	0.884	33532361	0.839
BJLY3_3	45728019	4924260	40803759	0.892	38657992	0.845
OH_1	40412793	6190652	34222141	0.847	32779302	0.811
OH_2	40913870	6389357	34524513	0.844	33109646	0.809
OH_3	42611518	5567003	37044515	0.869	35699008	0.838
XY4_1	34645083	2995489	31649594	0.914	29030189	0.838
XY4_2	37713396	3313356	34400040	0.912	32056160	0.85
XY4_3	36568438	3354418	33214020	0.908	30850599	0.844

Table S3. Sequences of gene-specific primers for transcriptome data and qPCR detection

Target gene	Primer	Sequence of gene-specific primer
Potri.013G057500	Potri.013G057500-F	5'-TTGGCTATCGTGGTTGTGCT-3'
	Potri.013G057500-R	5'-CACAAAAGTGGTGGCATCGG-3'
Potri.003G136700	Potri.003G136700-F	5'-GAGACCATGCCTGAGTGGAG-3'
	Potri.003G136700-R	5'-AAGGCTCCCATGGCAAACTT-3'
Potri.009G052500	Potri.009G052500-F	5'-GAGCCACAGAGGGAGTCAAC-3'
	Potri.009G052500-R	5'-AATGGAGTAGGCAGCAGCAG-3'
Potri.010G132000	Potri.010G132000-F	5'-CAAAGTTCCGGCCTCGAAAC-3'
	Potri.010G132000-R	5'-CTTGACCCCGATGCCTTCTT-3'
Potri.012G031100	Potri.012G031100-F	5'-CCCAAAAGGAGATTCCGGCT-3'
	Potri.012G031100-R	5'-TCGATTCCAGGCCAAACGAA-3'
Potri.002G146300	Potri.002G146300-F	5'-GGAGACACTGGATGCGACAA-3'
	Potri.002G146300-R	5'-ACACCCCATAGGCAAACCAG-3'
Potri.002G180800	Potri.002G180800-F	5'-GCAGCAGCATGAGTGATTCG-3'
	Potri.002G180800-R	5'-GTGAGAAGCTCTGGGGCAAT-3'
Potri.011G087200	Potri.011G087200-F	5'-TCAAGTTCTTCCCGGATGCC-3'
	Potri.011G087200-R	5'-CTGAGGCAAGCAATCCAGGA-3'
PtActin	PtActin-F	5'-TCATCGGAATGGAAGCTGCTGGTA-3'
	PtActin-R	5'-TAGTGGAACCACCACTGAGCACAA-3'

Table S4. Genetic expression patterns of plant hormone signal transduction pathway genes and TFs in hybrids

Pathway genes and TFs	Genetic expression patterns in Hyb1	Genetic expression patterns in Hyb2
ARR9	VIII	VIII
AUX1	VIII	/
WOL	VIII	/
BRI1-1	VIII	/
HAI2	VIII	II
ARR3	V	II
IAA16	II	/
BRI1-2	/	/
NPR3	/	/
LHY-1	II	VIII
LHY-2	II	II
TCP4	/	/
MYB88	VIII	/
NPR1	VII	/
BKI1	VII	/
MYC2	/	/

Note: II, high-paternal expression dominance; V, transgressive up-regulation; VII, transgressive down-regulation; VIII, transgressive up-regulation; /, genes don't belong to any genetic expression patterns in hybrids.

Figure Legends

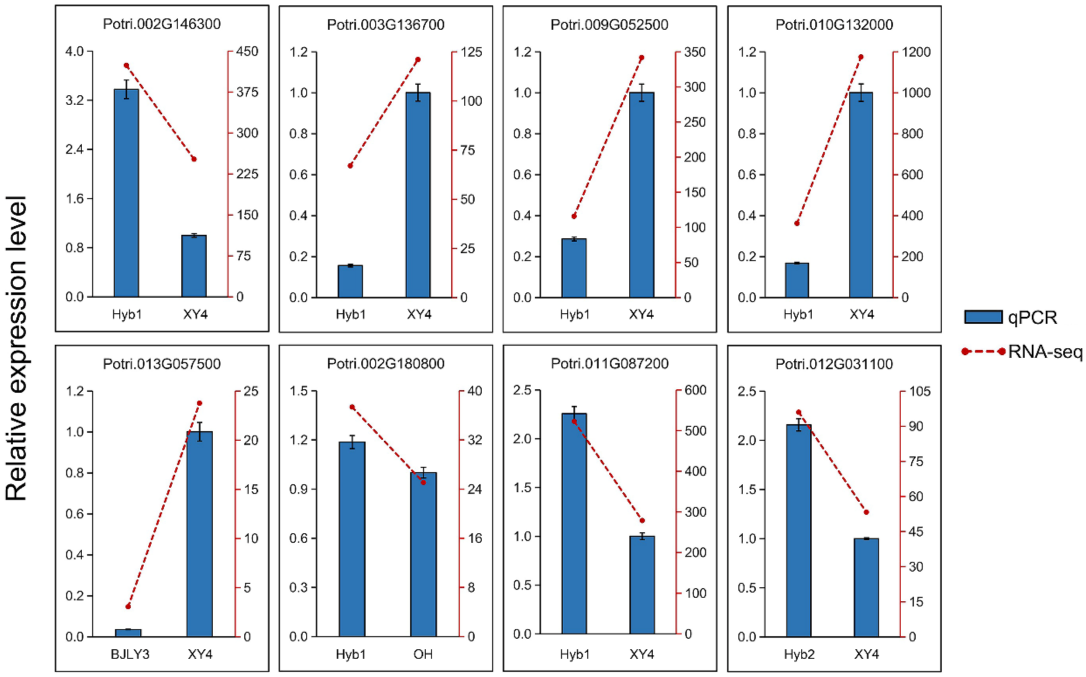

Figure S1. qPCR validation of the transcriptome data.

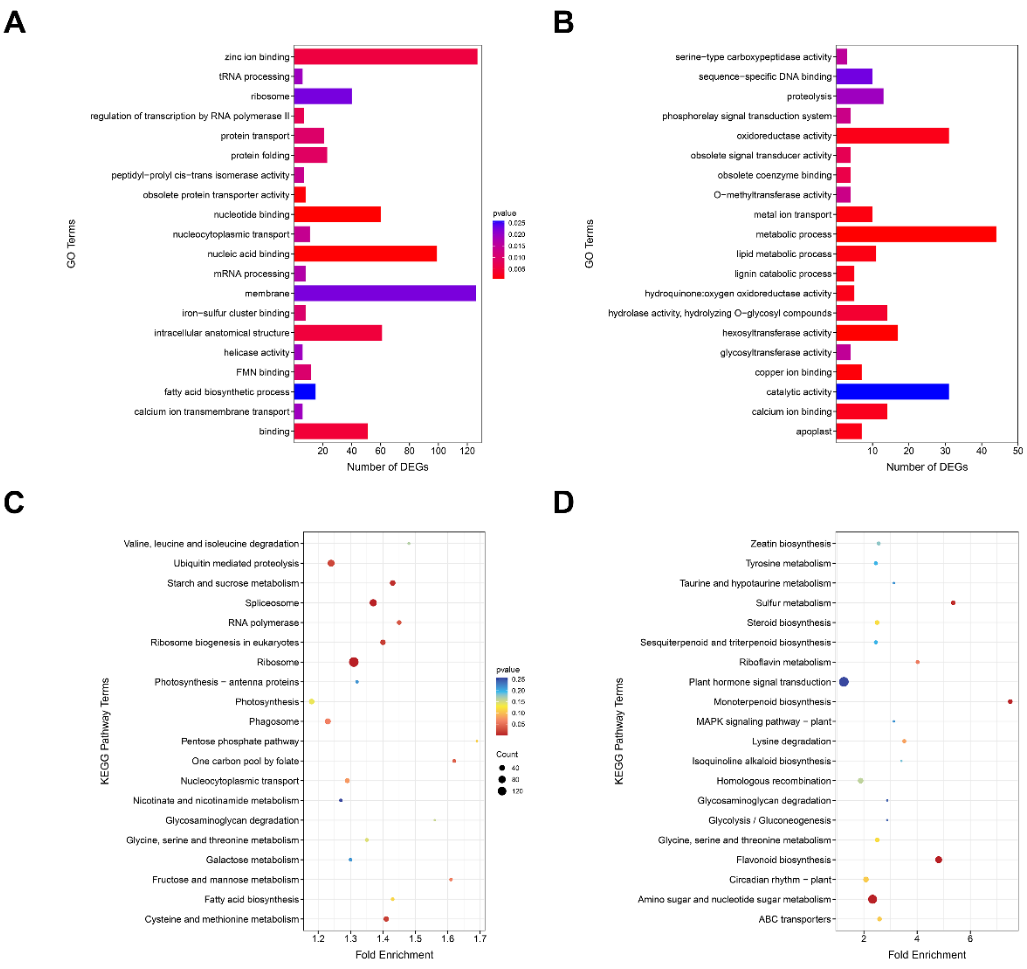

Figure S2. The plots of GO and KEGG enrichment analysis Hyb2. A–B The top 20 GO terms of dominance and over-dominance, respectively. C–D The top 20 KEGG pathways of dominance and over-dominance, respectively.
